# M2 Muscarinic Receptor-Dependent Contractions of Airway Smooth Muscle are Inhibited by Activation of β-Adrenoceptors

**DOI:** 10.1093/function/zqac050

**Published:** 2022-09-26

**Authors:** Tuleen Alkawadri, Pei Yee Wong, Zhihui Fong, Fionnuala T Lundy, Lorcan P McGarvey, Mark A Hollywood, Keith D Thornbury, Gerard P Sergeant

**Affiliations:** Smooth Muscle Research Centre, Dundalk Institute of Technology, Dublin Road, Dundalk, Co. Louth A91 K584, Ireland; Smooth Muscle Research Centre, Dundalk Institute of Technology, Dublin Road, Dundalk, Co. Louth A91 K584, Ireland; Smooth Muscle Research Centre, Dundalk Institute of Technology, Dublin Road, Dundalk, Co. Louth A91 K584, Ireland; The Wellcome-Wolfson Institute for Experimental Medicine, School of Medicine, Dentistry and Biomedical Sciences, Queen’s University Belfast, Belfast, BT9 7BL, Northern Ireland; The Wellcome-Wolfson Institute for Experimental Medicine, School of Medicine, Dentistry and Biomedical Sciences, Queen’s University Belfast, Belfast, BT9 7BL, Northern Ireland; Smooth Muscle Research Centre, Dundalk Institute of Technology, Dublin Road, Dundalk, Co. Louth A91 K584, Ireland; Smooth Muscle Research Centre, Dundalk Institute of Technology, Dublin Road, Dundalk, Co. Louth A91 K584, Ireland; Smooth Muscle Research Centre, Dundalk Institute of Technology, Dublin Road, Dundalk, Co. Louth A91 K584, Ireland

**Keywords:** airways, cholinergic, COPD, smooth muscle, muscarinic, bronchodilation

## Abstract

Beta-adrenoceptor (β-AR) agonists inhibit cholinergic contractions of airway smooth muscle (ASM), but the underlying mechanisms are unclear. ASM cells express M3 and M2 muscarinic receptors, but the bronchoconstrictor effects of acetylcholine are believed to result from activation of M3Rs, while the role of the M2Rs is confined to offsetting β-AR-dependent relaxations. However, a profound M2R-mediated hypersensitization of M3R-dependent contractions of ASM was recently reported, indicating an important role for M2Rs in cholinergic contractions of ASM. Here, we investigated if M2R-dependent contractions of murine bronchial rings were inhibited by activation of β-ARs. M2R-dependent contractions were apparent at low frequency (2Hz) electric field stimulation (EFS) and short (10s) stimulus  intervals. The β1-AR agonist, denopamine inhibited EFS-evoked contractions of ASM induced by reduction in stimulus interval from 100 to 10 s and was more effective at inhibiting contractions evoked by EFS at 2 than 20 Hz. Denopamine also abolished carbachol-evoked contractions that were resistant to the M3R antagonist 4-DAMP, similar to the effects of the M2R antagonists, methoctramine and AFDX-116. The inhibitory effects of denopamine on EFS-evoked contractions of ASM were smaller in preparations taken from M2R ^−/−^ mice, compared to wild-type (WT) controls. In contrast, inhibitory effects of the β3-AR agonist, BRL37344, on EFS-evoked contractions of detrusor strips taken from M2R ^−/−^ mice were greater than WT controls. These data suggest that M2R-dependent contractions of ASM were inhibited by activation of β1-ARs and that genetic ablation of M2Rs decreased the efficacy of β-AR agonists on cholinergic contractions.

## Graphical Abstract

**Graphical Abstract:**
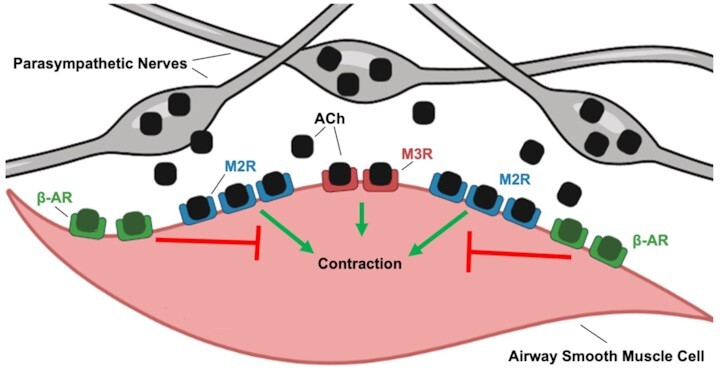

## Introduction

β2-adrenoceptor (β2-AR) agonists and muscarinic receptor (MR) antagonists are the mainstay bronchodilator treatments used to prevent, or alleviate the symptoms of, obstructive lung conditions such as COPD and asthma.^1^ β2-AR agonists target β2-ARs on airway smooth muscle (ASM) cells to induce ASM relaxation and reduce airway resistance,^2^ whereas MR antagonists prevent the bronchoconstrictor effects of acetylcholine (ACh) released from parasympathetic nerves.^3^ There is also evidence of “cross-talk” between the beta-adrenoceptor (β-AR) and MR signalling pathways in ASM cells,^4–7^ and it has been shown that β-AR agonists inhibit contractile responses induced by MR agonists.^8–11^ β-ARs are coupled to Gs-proteins, which activate adenylate cyclase to elevate cytosolic cAMP levels and stimulate protein kinase A (PKA), while the bronchoconstrictor effects of ACh are thought to result from activation of postjunctional M3Rs, even though they are outnumbered by M2Rs by a ratio of 4:1 in most species.^12–14^ The role of the postjunctional M2Rs in ASM is less clear.

The inhibitory effects of β-AR agonists on ASM contraction are potentiated by M2R antagonists, presumably by preventing activation of G_i_-proteins that decrease adenylate cyclase activity and would counteract the effects of β-AR activation.^6,10,12^,^15–18^ Therefore, it is thought that the main role of M2Rs in ASM is to provide a functional antagonism to the inhibitory effects of β-AR activation, rather than directly contributing to contractions induced by neurally released ACh. Nevertheless, studies on small airways of M3R knockout mice showed that cholinergic responses were ∼60% lower than wild-type (WT) controls, but were absent in mice that had both M2 and M3Rs knocked out, consistent with a direct role for M2Rs in cholinergic contractions of ASM.^19^ In addition, a profound M2R-mediated hypersensitization of M3R-dependent contractions of murine ASM was reported by Alkawadri et al.,^20^ indicating that activation of postjunctional M2Rs could make a greater contribution to cholinergic nerve-mediated contractions of ASM than previously realized.^21^

β-AR agonists are thought to exert their inhibitory effects on cholinergic contractions of ASM via inhibition of M3R signalling pathways. However, it has been noted that this assertion is based on sensitivity of known M3R-dependent pathways to PKA, rather than direct evidence showing that M3R signalling is affected by β-ARs in ASM cells.^4,5^ The finding that M2Rs make a direct contribution to ACh-dependent contractions of ASM raises the possibility that β-AR agonists could affect M2R-dependent contractions. In the present study, we found that the β-AR agonist denopamine inhibited M2R-dependent potentiation of electric field stimulation (EFS)-evoked contractions of ASM and abolished 4-DAMP-resistant contractions induced by CCh. Furthermore, the inhibitory effects of denopamine on EFS-induced contractions were reduced, rather than enhanced, in M2R null mice. These data suggest that M2R-dependent contractions of ASM are regulated by activation of β-ARs.

## Material and Methods

### Tissue Dissection

All procedures were carried out in accordance with current EU legislation and with the approval of Dundalk Institute of Technology Animal Use and Care Committee. Male and female C57BL/6 WT and B6N.129S4(Cg)-Chrm2^tm1Jwe^/J (M2R knock out) mice aged 10–16 wk old were humanely killed by intraperitoneal injection of pentobarbitone (100 mg/kg) and the lungs were removed and placed in oxygenated Krebs solution. M2R knock out (M2R KO) mice were purchased from the Jackson Laboratory (United States). Homozygote M2R KOs were generated from the breeding of heterozygotes and were identified by genotyping using PCR as per JAX protocols and reagents. The bronchial tree was exposed by sharp dissection under a microscope to remove surrounding blood vessels and lung tissue.^22^ The left and right bronchi were removed and cut into rings and placed in Krebs solution.

### Isometric Tension Recordings

Rings  (1-2 mm) from the right and left main bronchi were mounted in water-jacketed organ baths, perfused with warmed Krebs solution, adjusted to 5 m n tension, and equilibrated for 40 min. Isometric contractions, were recorded using a Myobath system, and data acquired using DataTrax2 software (World Precision Instruments). Mean contraction amplitude was measured by averaging peak contraction amplitude of 10 electric-field stimulation (EFS)-induced contractions during each parameter, or before and during drug addition (when they had their maximal effect). Drugs were added directly to the organ bath, where they were diluted in Krebs solution to their final concentration. Electrical field stimulation was used to excite transmural nerves and was applied via two platinum electrode wires (5 mm length, 2.5 mm apart) by a MultiStim system-D330 stimulator (Digitimer Ltd, England), which delivered trains of pulses (pulse amplitude 20 V, nominal; pulse width 0.3 ms; 1 or 10 s duration) at frequencies of 2, 4, or 20 Hz, at intervals of 10 or 100 s. Experiments, which compared responses in M2R KO mice with WT controls were performed at the same time.

### Drugs and Solutions

Carbachol, methoctramine, and BRL-37344 (Sigma Aldrich), indomethacin (Abcam), denopamine (Santa Cruz), and α,β-methylene ATP (Cayman) were dissolved in DMSO, ethanol, or distilled water as appropriate. Krebs solution was composed of: (m m) 120 NaCl, 5.9 KCl, 25 NaHCO_3_, 1.2 NaH_2_PO_4_·2H_2_O, 5.5 glucose, 1.2 MgCl_2_, and 2.5 CaCl_2_. pH was adjusted to 7.4 by bubbling the solution with 95% O_2_,−5% CO_2_.

### Data Analysis and Statistics

Experimental series were obtained from four or more animals; *n* refers to the number of tissue strips studied and *N* to the number of animals. Data were analyzed using Prism software (GraphPad). Summary data are presented as mean ± SEM. Statistical comparisons were performed on original (non-normalized) data using either Student’s paired *t*-test or, if three experimental groups were compared, ANOVA followed by Bonferonni post hoc test, with *P <*.05 considered statistically significant.

## Results

**Figure 1.**
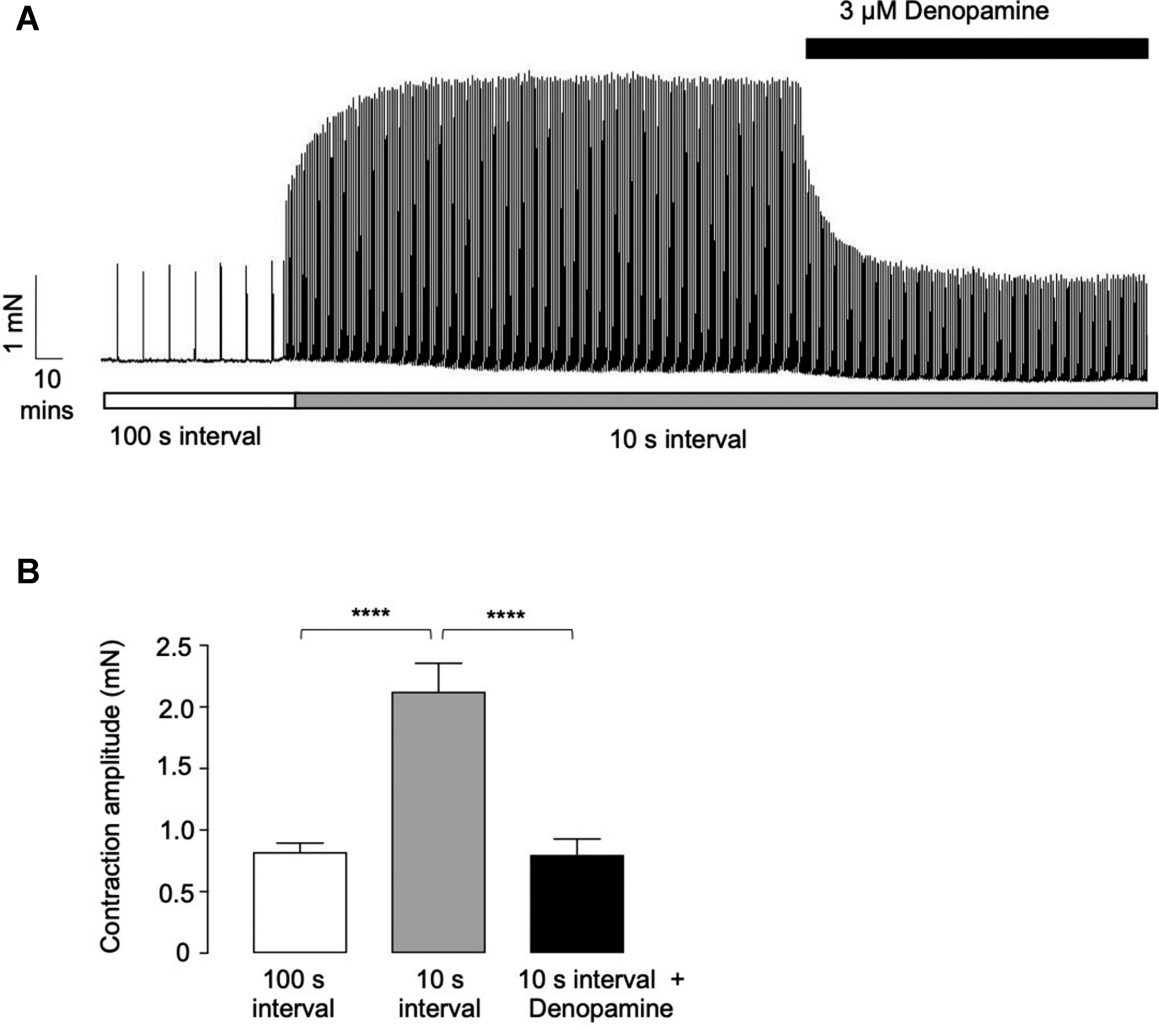
*Effects of the β1-AR agonist denopamine on EFS-evoked contractions of murine bronchial rings*. (A) is a representative isometric tension recording showing the effect of denopamine (3 µm) on EFS-evoked contractions (2 Hz) that were augmented by a reduction in stimulus interval from 10 to 100 s. Contraction amplitude was reduced to levels observed prior to reduction in stimulus interval. (B) is a summary bar chart showing the effect of denopamine on mean contraction amplitude, before and during the presence of denopamine (*n* = 15, *N* = 9, and ****P< .0001 ANOVA).

Alkawadri et al. demonstrated that the amplitude of EFS-evoked contractions of murine ASM were enhanced by a reduction in the stimulus interval from 100 to 10 s.^20^ This effect was inhibited by the M2R antagonists, methoctramine, and AFDX-116, and was absent in preparations taken from M2R null mice. Application of the M3R antagonist abolished the entire response.^20^ These data indicated that M3R-dependent contractions of ASM were potentiated by activation of postjunctional M2Rs. The purpose of the present study was to investigate if the M2R-dependent component of cholinergic contractions of murine ASM was affected by activation of β-ARs. Previous studies found that the inhibitory effects of β-AR agonists in murine ASM were brought about by activation of β1-ARs, and not β2-ARs as is the case in humans.^23,24^ For example, Birrell et al. showed that relaxations of murine trachea induced by the nonselective β-adrenoceptor agonist, isoprenaline, were inhibited by genetic ablation of β1-AR, but not β2-ARs.^24^ Therefore, we examined if denopamine, a selective β1-AR agonist, inhibited the M2R-dependent potentiation of EFS-evoked contractions of ASM induced by reduction in stimulus interval. Figure 1(A) shows a representative trace demonstrating that reducing the stimulus interval from 100 to 10 s enhanced contraction amplitude, and that this effect was reversed by denopamine (3 μm). In 15 preparations, mean contraction amplitude increased from 0.8 ± 0.07 to 2.1 ± 0.2 m n, following reduction in stimulus interval and decreased to 0.8 ± 0.1 m n (*P* < .0001, ANOVA, *n* = 15, and *N* = 9) when denopamine was added.

**Figure 2.**
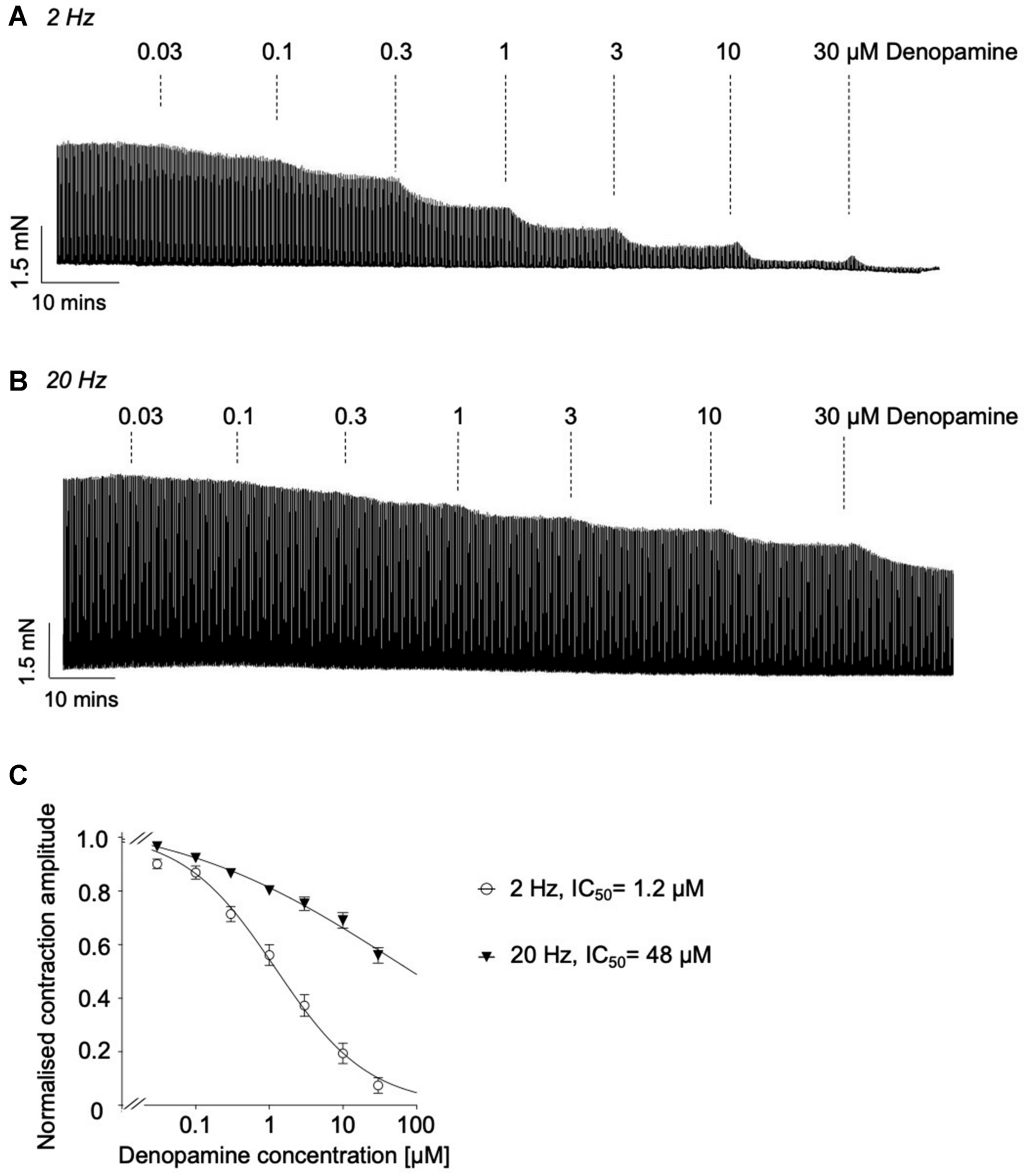
Comparison of the effects of increasing concentrations of denopamine on contractions of murine bronchial rings evoked by EFS at 2 and 20 Hz. (A) and (B) are representative isometric tension recordings showing the effect of cumulative addition of denopamine (30 n m to 30 µm) on contractions by EFS at 2 and 20 Hz, respectively (10 s intervals). (C) Summary concentration-effect curves for denopamine on contractions evoked by EFS at 2 Hz (circles, IC_50_ = 1.2 µm, *n* = 17, and *N* = 9) and 20 Hz (triangles, IC_50_ = 48 µm, *n* = 8, and *N* = 5), respectively.

M2R-dependent potentiation of ASM contractions was evident at 2 Hz EFS, but not 20 Hz.^20^ Therefore, to further examine if β-AR activation affected the M2R-component of the response we compared the effects of denopamine on contractions evoked at both frequencies. The representative traces in Figure 2(A) and (B) show the effect of cumulative addition of denopamine, over the concentration range 0.03–30 μm, on contractions induced by EFS at 2 and 20 Hz, respectively. It was clear that contractions evoked by EFS at 2 Hz were more sensitive to denopamine than those at 20 Hz. This is borne out in the summary concentration-effect curves in Figure 2(C), which indicate that denopamine inhibited responses evoked by 2 Hz EFS with an IC_50_ of 1.2 μm (95% CI: 0.86 −1.73 μm, *n* = 17, and *N* = 9) versus 48 μm (95% CI: 27.2–86.4 μm, *n* = 8, and *N* = 5) for responses evoked by 20 Hz EFS.

**Figure 3.**
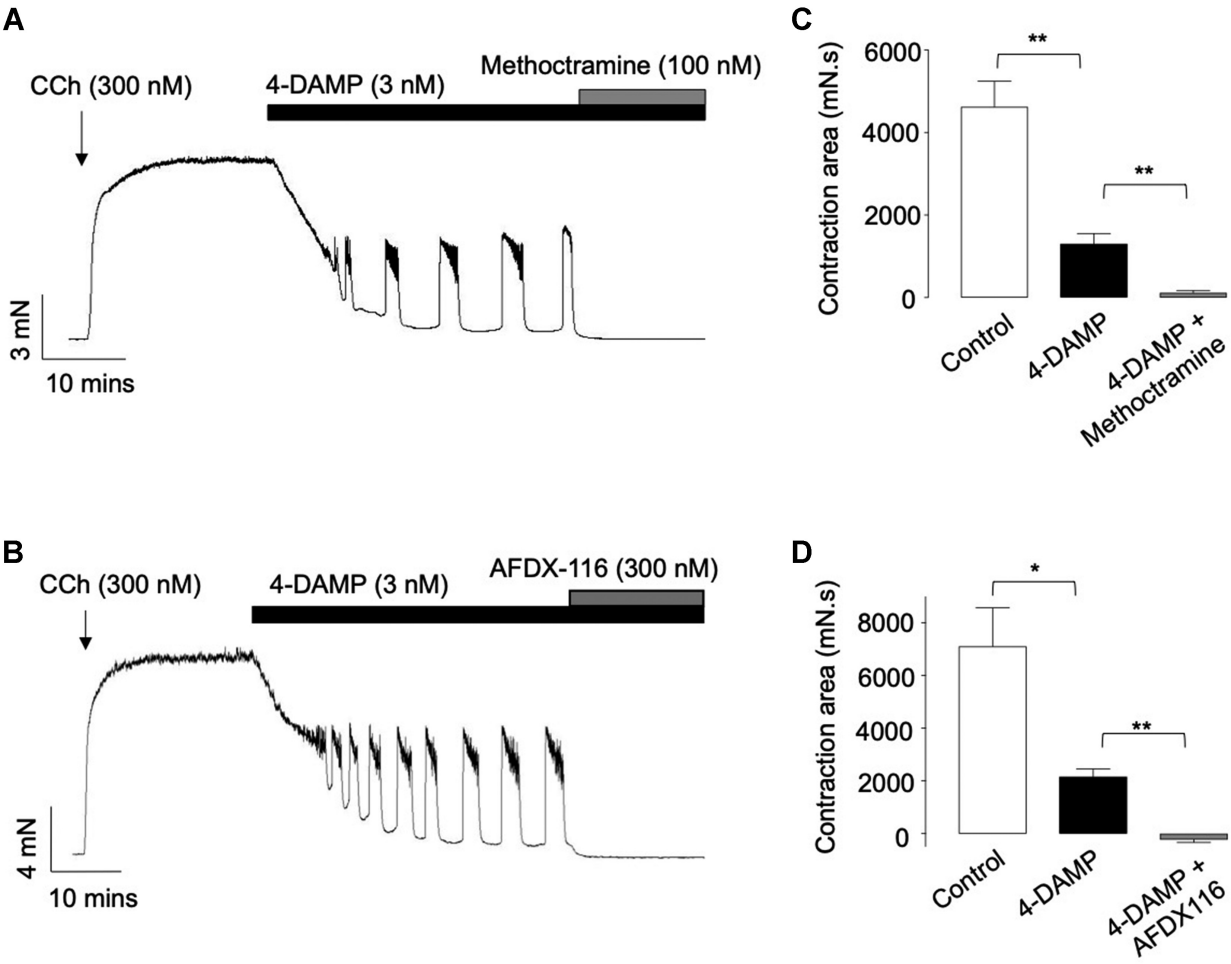
Effects of M2R antagonists methoctramine and AFDX-116 on CCh-evoked contractions of murine bronchial rings in the presence of 4-DAMP. (A) and (B) are isometric tension recording showing that methoctramine (100 n m; A) and AFDX-116 (300 n m; B) abolished CCh-induced contractions that remained in the presence of 4-DAMP (3 n m). (C) is a summary bar chart showing mean contraction amplitude (measured by area under the curve) before and during the presence of 4-DAMP and 4-DAMP plus methoctramine (*n* = 9, *N* = 9, **P< .01, ANOVA). (D) is a summary bar chart showing mean contraction amplitude before and during the presence of 4-DAMP, and 4-DAMP plus AFDX-116 (*n* = 6, *N* = 4, **P < .01, *P < .05, ANOVA).

The representative traces in Figure 3(A) and (B) show that CCh-evoked contractions of ASM were reduced by the selective M3R antagonist, 4-DAMP (3 n m), resulting in a series of oscillatory contractions. These 4-DAMP-resistant contractions were abolished by the selective M2R antagonists methoctramine (100 n m, Figure 3A) and AFDX-116 (300 n m, Figure 3B), respectively. Summary data in Figure 3(C) show that 4-DAMP reduced the amplitude of CCh-induced contractions by 72% and that the amplitude of the residual contractions were reduced to less than 1% of control in the presence of 4-DAMP and methoctramine (*n* = 9, *N* = 9, *P* < .01, ANOVA). Similar results were achieved with AFDX-116 ( *n* = 6, *N* = 4, *P*< .01, ANOVA,   Figure 3D). Therefore, it was apparent that the 4-DAMP-insensitive contractions, evoked by 300 n m CCh, resulted from activation of M2Rs and we next examined if these contractions were sensitive to denopamine treatment.

**Figure 4.**
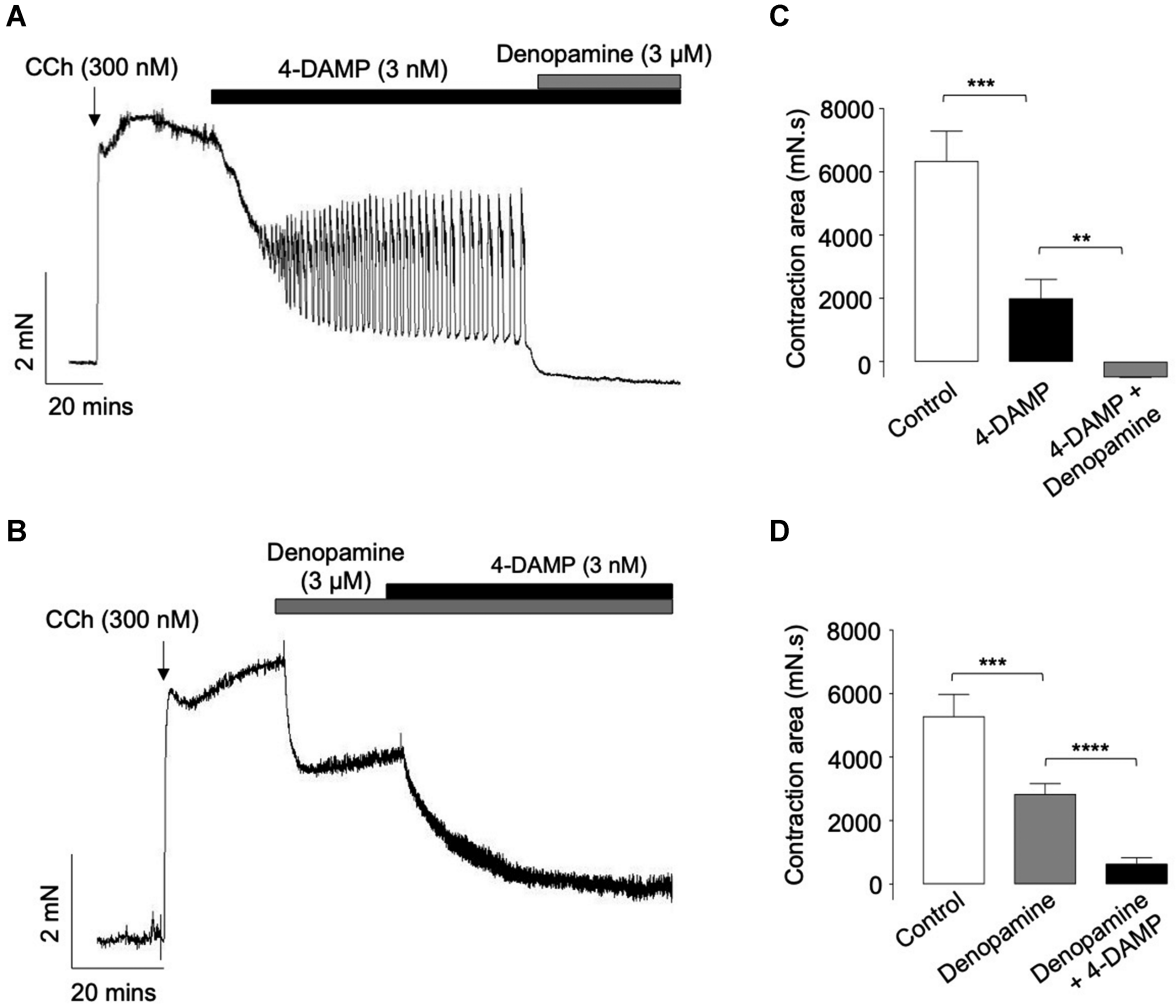
*Effects of denopamine on CCh-evoked contractions of murine bronchial rings, before and during the presence of 4-DAMP*. (A) is an isometric tension recording showing that denopamine (3 µm) abolished CCh-induced contractions that remained in the presence of 4-DAMP (3 n m). (B) is an isometric tension recording showing that denopamine (3 µm) reduced the amplitude of CCh-induced contractions of ASM and that the residual contractions were inhibited by 4-DAMP (3 n m). (C) is a summary bar chart showing mean contraction amplitude (measured by area under the curve) before and during the presence of 4-DAMP and 4-DAMP plus denopamine (*n* = 7, *N* = 6, ***P< .001, and **P< .01, ANOVA). (D) is a summary bar chart showing mean contraction amplitude before and during the presence of denopamine, and denopamine plus 4-DAMP (*n* = 16, *N* = 8, ***P < .001, and ****P< .0001, ANOVA).

The representative trace in Figure 4(A) shows that the oscillatory contractions that remained in the presence of 4-DAMP (3 n m) were abolished by denopamine (3 μm), indicating that the M2R component of the response was abolished by activation of β1-ARs. Summary data in Figure 4(C) indicate that 4-DAMP reduced the contractions by 68% (*n* = 7, *N* = 6, and *P*< .001) and that subsequent addition of denopamine (3 μm) abolished the remaining contractions (*P*< .01 ) and reduced resting tone below that recorded prior to CCh application. In contrast, when denopamine was added first, before 4-DAMP, it reduced the amplitude of the CCh response by 46% (*P*< .001, *n* = 16, and *N* = 8). Subsequent addition of 4-DAMP greatly reduced the amplitude of the denopamine-resistant contraction (Figures 4B and D; *P*< .0001). Therefore, denopamine abolished 4-DAMP-resistant contractions of ASM, but a large 4-DAMP-sensitive component of the CCh response remained when denopamine was added first. This indicates that denopamine abolished the M2R-component of the CCh response, but that a large M3R-component was unaffected by denopamine.

**Figure 5.**
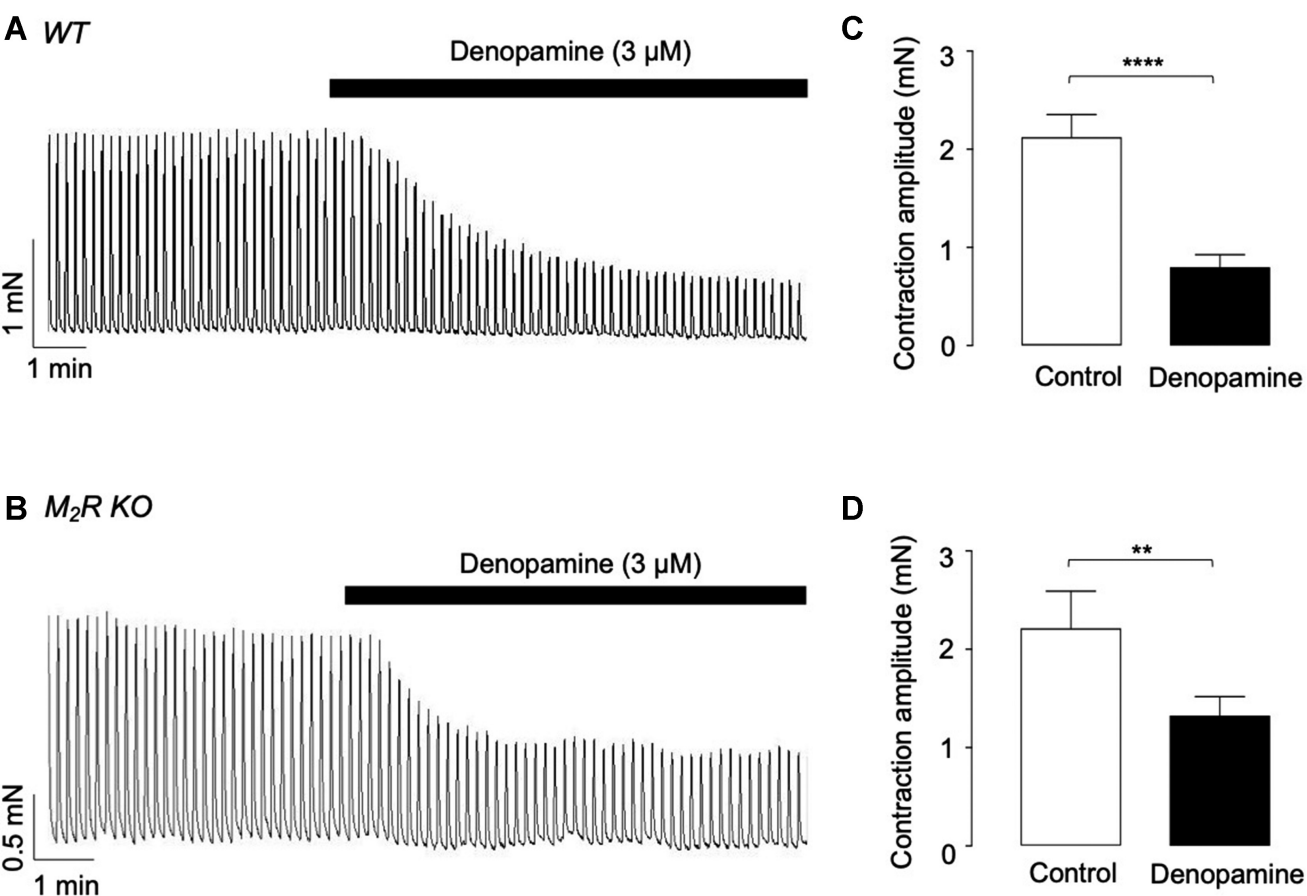
Comparison of the effects of denopamine on EFS-evoked contractions of ASM taken from WT and M2R null mice. (A) and (B) are representative isometric tension recordings showing the effect of 3 µM denopamine on EFS-evoked contractions (2 Hz), taken from WT and M2R null mice, respectively. (C) and (D) are summary bar charts showing the effect of denopamine (3 µm) on mean contraction amplitude in WT (*n* = 15, *N* = 9, and ****P< .0001, and two-tailed Student’s *t*-test) and M2R null mice (*n* = 9, *N* = 5, and **P < .01, and two-tailed Student’s *t*-test).

Previous studies indicated that β-AR agonists inhibited cholinergic contractions of ASM via PKA-mediated inhibition of M3R-dependent signalling pathways.^4^ In addition, it has been suggested that postjunctional M2Rs are not functionally involved in cholinergic contractions of ASM, but instead provide a functional antagonism to β-AR activation.^15–18^ If this model were correct, then blockade, or removal, of M2Rs would be expected to enhance the inhibitory effects of β-AR agonists, since the opposing effects of M2R activation would be lost. We investigated if this was the case in ASM by comparing the effects of denopamine on cholinergic nerve-evoked contractions taken from WT and M2R null mice. Representative traces showing the effect of denopamine on EFS-evoked contractions of ASM from WT and M2R null mice are shown in Figure 5(A) and (B) and corresponding summary data, are provided in Figure 5(C) and (D), respectively. In 9 WT mice, denopamine reduced mean contraction amplitude by 62% from 2.1 ± 0.2 to 0.8 ± 0.1 m n (*P* < .0001, and *n* = 15), whereas in M2R null preparations contractions were only reduced by 40.9% (from 2.2 ± 0.2 to 1.3 ± 0.1 m n, *P* < .01, *n* = 9, *N* = 5). Therefore, the effects of denopamine on EFS responses were reduced, rather than enhanced, in preparations lacking M2Rs. These data are at odds with the current models, which suggest that only role of M2Rs in ASM is to provide a functional antagonism to β-AR activation.

**Figure 6.**
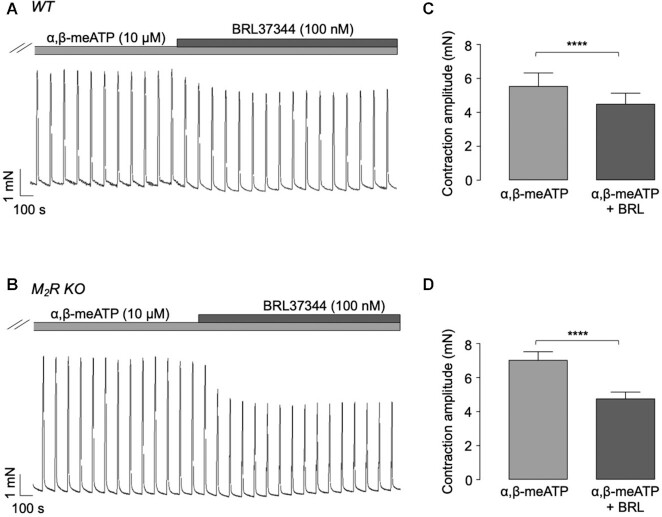
Comparison of the effects of the β3-AR agonist BRL37344 on EFS-evoked contractions of detrusor strips taken from WT and M2R null mice. (A) and (B) are representative isometric tension recordings showing the effect of 100 n m BRL37344 on EFS-evoked contractions (4 Hz) of detrusor strips taken from WT and M2R null mice, respectively. (C) and (D) are summary bar charts showing the effect of BRL37344 on mean contraction amplitude in WT (*n* = 16, *N* = 8, and ****P < .0001, two-tailed Student’s paired *t*-test) and M2R null mice (*n* = 16, *N* = 8, and **** *P*< .0001, and two-tailed Student’s paired *t*-test).

Functional antagonism between M2R and β-AR activation has been observed in studies of detrusor smooth muscle.^25^ Therefore, we examined if the β3-AR agonist, BRL37344 (100 n m) induced larger inhibitory effects on EFS-evoked contractions in detrusor strips from M2R null mice, compared to WT. These experiments were performed in the presence of α,β-methylene ATP (10 μm) to desensitize P2X1 receptors and isolate the cholinergic component of the EFS response. In contrast to the effects of denopamine on ASM, BRL37344 (100 n m) induced greater inhibitory effects in bladder strips taken from M2R null mice (Figure 6B) compared to WT mice (Figure 6A). Summary data in Figure 6(C) and (D) show that BRL37344 reduced mean contraction amplitude by 18.7% in WT mice (5.6 ± 0.8 to 4.5 ± 0.6 m n, *n* = 16, *N* = 8) compared to 32.2% (7 ± 0.5 to 4.8 ± 0.4 m n, *n* = 16, and *N* = 8) in M2R KOs.

## Discussion

Airway diameter is regulated by contraction of ASM and enhanced ASM contraction leads to increased airway resistance, a feature of obstructive airway conditions such as COPD.^26,27^ Increased cholinergic tone is the main reversible component of airway obstruction in COPD,^28–31^ therefore, it is of interest to understand the mechanisms underlying cholinergic contractions of ASM and how they are targeted by β-AR agonists. Most studies have determined that cholinergic tone of ASM results from activation of postjunctional M3Rs on ASM cells and is regulated by activation of autoinhibitory M2Rs located on parasympathetic nerves that limit ACh output.^32,33^ The contribution of postjunctional M2Rs to cholinergic tone of ASM is less clear and most studies conclude that their contribution to ACh-induced contractions is negligible, but that their activation can counteract inhibitory effects of β-AR agonists.^10,12,14,16,18^

The findings of the present study demonstrate that activation of β1-ARs reduces the amplitude of cholinergic contractions of ASM and that this effect involves inhibition of M2R-dependent responses. In addition, we found that the inhibitory effects of the β1-AR agonist denopamine on contractions of ASM induced by cholinergic stimuli in M2R KO mice were reduced and not enhanced. Therefore, contrary to previous studies, we suggest that M2Rs are functionally involved in cholinergic contractions of ASM and that inhibition of this response contributes to the inhibitory effects of β-AR agonists on the bronchoconstrictor effects of ACh. Consequently, our understanding of the mechanisms responsible for contraction of ASM by ACh and its regulation by β-AR agonists should be reviewed.

Alkawadri et al.^20^ demonstrated that the amplitude of M3R-dependent contractions of ASM were augmented by activation of M2Rs. M2R-dependent contractions were revealed by EFS at low frequency (2 Hz) and short stimulus intervals (10 s).^20^ Here, we show that the inhibitory effects of M2R antagonists on these responses, were mimicked by application of the β1-AR agonist, denopamine. In addition, denopamine also inhibited 4-DAMP-resistant contractions of ASM evoked by CCh, consistent with the idea that M2R-dependent contractions of ASM are inhibited by β-AR activation. Previous studies suggested that there was crosstalk between β-AR and M3R signalling pathways in ASM cells,^7^ but our results suggest that there may also be an interaction between β-AR and M2R pathways.

The degree to which inhibition of M2R-dependent responses contributes to the overall inhibitory effect of β-AR agonists on cholinergic contractions of ASM is likely to depend on a combination of the relative contribution of M2R and M3Rs to the cholinergic response and on the relative sensitivity of the M3 and M2R components to β-AR activation. The former is highly dependent on the stimulus parameters and it seems likely from other studies that the M2R component is more prevalent at low ACh concentrations.^34,35^ With regards to the relative sensitivity of the M2 and M3R responses, it was interesting to note that a large 4-DAMP-sensitive contraction remained in the presence of denopamine, suggesting that the M3R-dependent contractions was not fully blocked by β-ARs activation. In contrast, the 4-DAMP-resistant component of the CCh response was abolished by denopamine, suggesting that the entire M2R response was ablated by activation of β-ARs. Similarly, contractions evoked by 20 Hz EFS, known to involve M3, but not M2Rs, displayed a weaker sensitivity to denopamine than those induced by 2 Hz EFS, which contain a larger M2R-dependent component. Therefore, it is plausible that, at least in murine ASM, M2R-dependent signalling pathways have a greater sensitivity to activation of the β-AR/cAMP pathway than the M3R pathways.

The cellular mechanisms underlying M2R-dependent contractions of ASM have not been fully elucidated. However, Semenov et al. reported that M2R signalling in tracheal smooth muscle leads to depolarization and recruitment of voltage-operated Ca^2+^ channels, and that these responses are functionally opposed by activation of large conductance Ca^2+^-activated K^+^ (BK) channels.^34^ It has also been shown that activation of β-ARs in ASM leads to BK channel activation.^36,37^ Therefore, it is conceivable that β-AR agonists inhibit M2R-dependent responses by such a mechanism, leading to membrane hyperpolarization and reduced activation of voltage-operated Ca^2+^ channels. However, further investigation would be required to determine if this is the case.

The results of the present study support the concept of functional antagonism between activation of β-AR and M2Rs in the bladder, as reported previously,^25,38^ but not in the airways. It is unclear why this difference exists between both tissues, but it seems likely that a greater functional role for M2Rs in cholinergic contractions of ASM is a factor. If M2R responses are targeted by β-AR agonists to a greater degree than the M3R component, then tissues with more involvement of M2Rs in the cholinergic response would be expected to display a greater sensitivity to β-AR agonists. Consequently, ablation of M2Rs would result in reduced efficacy of β-AR agonists in these tissues. Our results are in agreement with findings from Matsui et al.,^38^ which showed that the inhibitory effects of isoprenaline on contractions of murine bladder induced by the cholinergic agonist oxotremorine-M were enhanced in M2R KO mice, but were unchanged in tracheal smooth muscle. They reasoned that part of the isoprenaline response in the airways was independent of cAMP, and was thus unaffected by reduction in cAMP levels following M2R activation. However, this explanation does not account for the large potentiation of β-AR effects induced by M2R antagonists in ASM, such as those reported by Brown et al.,^18^ which found that isoproterenol-induced relaxations of rat and human small airways were significantly increased by AFDX-116 and gallamine. Therefore, it appears that the effects of β-AR agonists on cholinergic responses in ASM are enhanced by M2R antagonists^10,12,18^ but are unaffected, or reduced, when M2Rs are genetically ablated.^38^ It is possible that this difference arises from compensatory changes in gene expression that may occur in M2R KO mice. However, based on the data presented here, this appears to be only a feature of ASM, and not bladder smooth muscle, and further investigation of this issue is required. It should also be noted that some studies suggest that enhanced effects of β-AR agonists in the presence of M2R antagonists, result from reduced levels of cholinergic tone and are not necessarily due to blockade of M2Rs.^39^ In the present study, it was notable that even though the mean amplitude of EFS-induced contractions was lower in the M2R knock-outs compared to WT preparations, the effects of denopamine were reduced, rather than enhanced. Therefore, the lack of functional antagonism observed in the present study does not appear to be due to a difference in the level of tone.

The therapeutic effects of anticholinergics, such as tiotropium, for asthma and COPD, are primarily thought to arise from blockade of M3Rs.^4^ Indeed, anticholinergics with a higher kinetic selectivity for M3Rs over M2Rs are preferred as they limit unwanted effects arising from inhibition of autoinhibitory M2Rs that would enhance ACh output.^40,41^ However, this logic fails to recognize the possibility that, under specific conditions, activation of M2Rs may contribute to the bronchoconstrictor effects of ACh.^20^ The results of the present study demonstrate that β-AR agonists inhibit the M2R-dependent component of the cholinergic response and it is possible that this may contribute to the therapeutic effects of these agents. If so, this may also contribute to the superior improvements in pulmonary function in patients taking a combination of β-AR agonists and M2R-sparing anticholinergics, than when either are administered alone.^42,43^ It is clear that further experimentation using human samples would be required to discern if this is the case. In addition, it should be noted that such experiments would require use of selective β2, rather than β1, adrenoceptor agonists.

In summary, we demonstrate that M2R-dependent contractions of ASM are inhibited by activation of β-ARs and that genetic ablation of M2Rs decreases the efficacy of the β-AR agonist denopamine on cholinergic contractions.

## Data Availability

The data underlying this article will be shared on reasonable request to the corresponding author.
